# Recent Advances in Additive Friction Stir Deposition: A Critical Review

**DOI:** 10.3390/ma17215205

**Published:** 2024-10-25

**Authors:** Xinze Dong, Mengran Zhou, Yingxin Geng, Yuxiang Han, Zhiguo Lei, Gaoqiang Chen, Qingyu Shi

**Affiliations:** 1 State Key Laboratory of Clean and Efficient Turbomachinery Power Equipment, Department of Mechanical Engineering, Tsinghua University, Beijing 100084, China; 2 Key Laboratory for Advanced Materials Processing Technology, Ministry of Education, Beijing 100084, China

**Keywords:** additive friction stir deposition, additive manufacturing, smart manufacturing, microstructure, mechanical property

## Abstract

Additive friction stir deposition (AFSD) is a novel solid-state additive manufacturing method developed on the principle of stirring friction. Benefits from its solid-phase properties, compared with traditional additive manufacturing based on melting–solidification cycles, AFSD solves the problems of porosity, cracks, and residual stress caused by the melting–solidification process, and has a significant improvement in efficiency. In AFSD, the interaction between feedstocks and high-speed rotating print heads suffers severe plastic deformation at high temperatures below the melting point, ending up in fine, equiaxed recrystallized grains. The above characteristics make components by AFSD show similar mechanical behaviors to the forged ones. This article reviews the development of AFSD technology, elaborates on the basic principles, compares the macroscopic formability and material flow behavior of AFSD processes using different types of feedstocks, summarizes the microstructure and mechanical properties obtained from the AFSD of alloys with different compositions, and finally provides an outlook on the development trends, opportunities, and challenges to the researchers and industrial fields concerning AFSD.

## 1. Introduction

In this section, firstly, a brief introduction is given for friction stir welding (FSW). Then, the development history and basic principles of additive friction stir deposition (AFSD) are introduced in detail. Finally, in order to highlight the unique advantages of additive friction stir deposition, a comparison is made between AFSD and traditional additive manufacturing (AM) methods.

### 1.1. Metal Welding by Solid-State Principle

**Figure 1:**
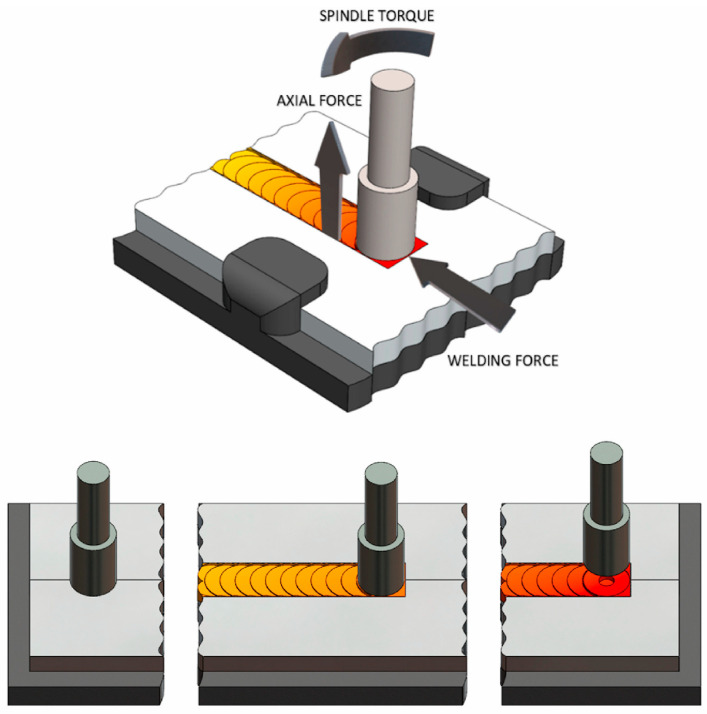
Schematic illustration of friction stir welding [1].

Friction stir welding (FSW) is a novel solid-state welding technique invented by The Welding Institute (TWI, UK) in 1991 and has already been widely used. As shown in Figure 1, during FSW, a high-speed rotating stirring tool with a shoulder and probe is inserted into the welding area and moves forward along the weld seam, introducing friction, compression, and shear effects. Under these effects, the surrounding material generates friction and deformation heat, and the material undergoes thermo-plasticization. Under the mutual effect of thermal and plastic flow, the previously separated weld seam is eliminated, forming a metallurgical bond in the weld seam area, and two previously separated components are bonded together.

### 1.2. Additive Manufacturing Technology Driven by Friction Stir Principle

**Figure 2:**
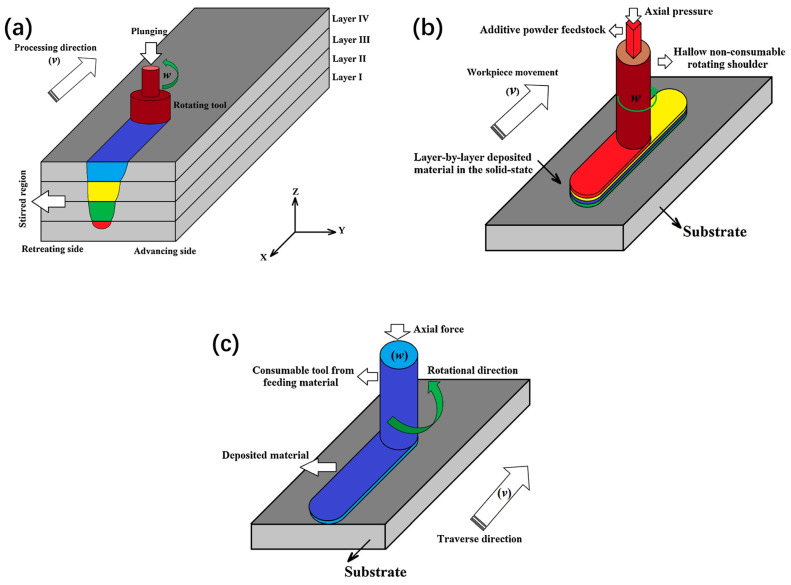
AM technology driven by friction stir principle [4]. (**a**) FSAM and (**b**,**c**) AFSD for non-consumable print head and consumable print head.

Given the unique solid-phase characteristics of FSW, researchers have applied the combined principle of friction and stir to the field of additive manufacturing (AM) and developed a new AM technique. According to its process characteristics, it can be roughly divided into two categories: The first one is friction stir additive manufacturing (FSAM), using stacking plates and performing friction stir welding to vertically connect the plates layer by layer (Figure 2a). The second kind is additive friction stir deposition (AFSD), which utilizes rods, wires, or powders as feedstock and deposits them through a hollow print head for deposition (Figure 2b), or directly uses consumable rod-shaped feedstock as a print head to achieve deposition (Figure 2c). The principles of the two categories are the same, but the manufacturing processes are different. The process route of FSAM and laminated object manufacturing is highly similar. FSAM uses prefabricated metal sheets, with a high-speed rotating stirring tool with a shoulder and stirring probe inserts into the sheets. The tool moves along a predetermined path, resulting in heat generation by friction and severe plastic deformation and leading to metallurgical bonding between the layers of the sheets [2,3] (Figure 2a). The metal sheets used in FSAM require good processability, which limits the available types of materials. In addition, customized sheets may be required for components of different sizes or shapes, which will reduce the universality of the process and may generate a large amount of waste during subtractive manufacturing after additive manufacturing, thereby increasing manufacturing costs.

The mechanism of AFSD is different from FSAM with the origin of feed materials. In the AFSD process, the feeding device applies feeding force to the feedstock, so that they are transported through a hollow shaft and rotated at a high speed with the print head. The dynamic friction and plastic deformation between the print head, the material to be deposited, and the previously deposited material (or substrate) cause the material to heat up and soften, and then the high temperature and plastic deformation promote the bonding of solid metals (Figure 2b). As the print head moves along a predetermined path, the metal material is repeatedly deposited layer by layer, and components with specific geometric shapes are manufactured. It is also possible to directly use consumable rodlike feedstock as a print head without using an additional print head, which generates friction heat and severe plastic deformation connected to the action of feeding force and rotation and achieves layer-by-layer deposition of materials (Figure 2c). In contrast, AFSD is more flexible in the selection of feedstock and generates less waste during subtractive manufacturing. In addition, AFSD also has more significant advantages in complex shape forming, spatial resolution, and free degree. Except for some special occasions, it can be seen that AFSD has stronger applicability and universality, with greater application prospects, and is more worthy of further research.

### 1.3. Comparison between Additive Friction Stir Deposition and Fusion-Based Additive Manufacturing

AM based on fusion generally requires high energy density heat sources such as laser, plasma beam, electron beam, or arc to melt the raw materials for additive manufacturing. Due to the involvement of melting–solidification cycles, AM based on fusion typically needs to be conducted in a gas-shielded or vacuum environment. In addition, the melting–solidification process may bring a series of problems, such as hot cracks, porosity, residual stress, etc. During the rapid melting and solidification of fusion-based metal AM, the melt pool conducts heat into the substrate, forming a curved solid–liquid interface [5]. During solidification, crystals grow competitively from the substrate or previously deposited layers [5]. In polycrystalline materials, competitive growth occurs among dendrites with different crystallographic orientations [6].

AFSD avoids the melting–solidification cycles, which not only avoids problems such as hot crack and porosity, but also can be carried out in an atmospheric environment with lower energy consumption, larger forming dimensions, and higher efficiency [7]. In addition, due to its microstructure mainly composed of fine equiaxed grains (similar to forged pieces), its mechanical properties are better. Given the above advantages, AFSD has enormous potential for application in fields such as automotive, transportation, navigation, and nuclear energy.

This review first introduced different types of AFSD processes, focusing on the most widely studied type (non-consumable print head and rodlike feedstock), then elaborated on the microstructure evolution, material flow, and overall properties. Finally, the development trend and potential opportunities and challenges faced by AFSD technology were discussed.

## 2. Methods

### 2.1. Strategy of Search and Selection

Relevant studies in the literature were searched using Google Scholar, ScienceDirect, Web of Science, and Springer, mostly focusing on representative studies from the past five years. Articles including the process methods, microstructure, and mechanical properties of AFSD were identified using the following keywords: “AFSD” OR “additive friction stir deposition” OR “additive friction stir” OR “additive friction” OR “FSAM” OR “friction stir additive manufacturing” OR “additive” OR “friction stir” OR “friction”. After the search, highly similar or identical studies were excluded; subsequently, studies were screened based on criteria of inclusion and exclusion.

### 2.2. Criteria of Inclusion and Exclusion

**Figure 3:**
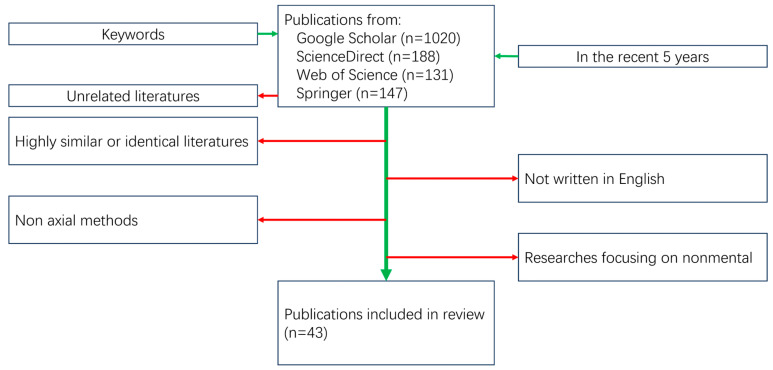
A flowchart of the selection process.

Only the original articles were considered; the works and results of these articles focused on or mainly involved process methods and parameters, microstructure, material flow, and mechanical properties of AFSD. The exclusion criteria are as follows: publications not written in English, research studies focusing on nonmetals, and any friction additive techniques using non-axial methods. The process of selection is shown in Figure 3.

## 3. Process

In terms of the process, AFSD can be roughly divided into two categories: the first uses a consumable print head as the feedstock directly, with some studies also calling it friction surfacing, and the second uses an extra print head to achieve the deposition of the feedstock. In the second category, the forms of feedstocks include rods, powders, wires, etc. This section introduces different categories of AFSD processes in detail. The focus is on comparing macroscopic formability, the behavior of material flow, defects, and microstructure, so their characteristics are highlighted.

### 3.1. Additive Friction Stir Deposition Using Consumable Print Head

**Figure 4:**
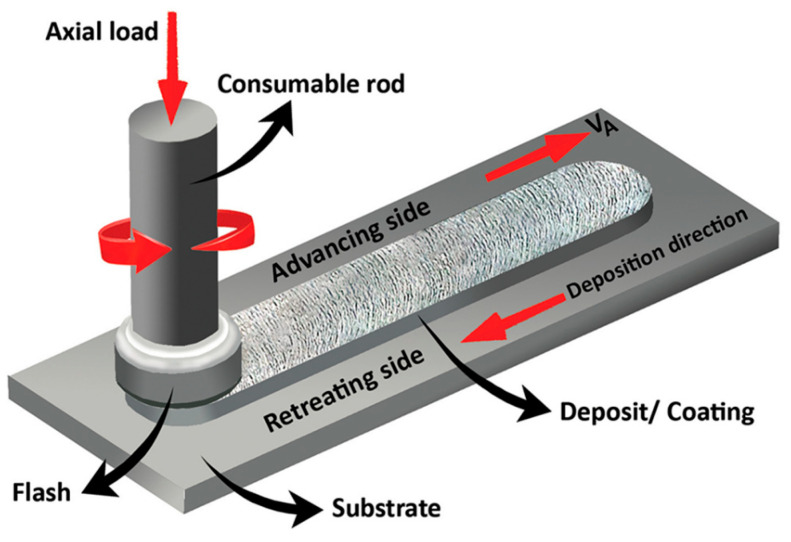
Schematic drawing of additive friction stir deposition using consumable print head [8].

This type of process directly uses consumable rodlike feedstock as a print head, thereby achieving additive deposition. As shown in Figure 4, during specific implementation, the high-speed rotating rodlike feedstock is directly in contact with the substrate, and a certain axial force is applied to the rod. The frictional heat causes the material to heat up, soften, and undergo severe plastic deformation. As the rod and substrate move laterally relative to each other, the consumable rodlike metal deposits onto the substrate.

**Figure 5:**
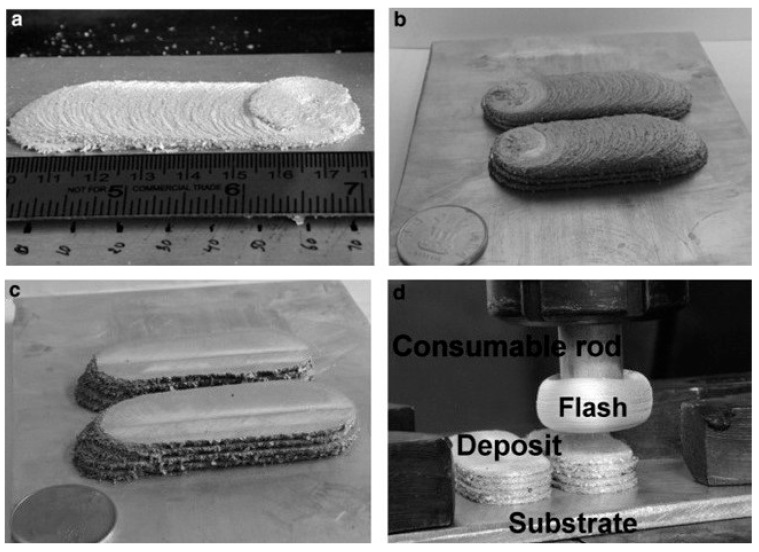
Several stages during the deposition: (**a**) one-layer and (**b**) three-layer depositions without top surface machining, (**c**) top surface-machined four-layer deposition, and (**d**) the buildings and the remaining feedstock after the fifth layer deposition [9].

Dilip et al. [9] used AA2014 aluminum alloys as the consumable print head and prepared additive deposits using AFSD. Figure 5 shows the appearance of several deposits. In Figure 5a–c, it can be seen that the surface quality of the deposits is poor and that the forming accuracy is low. Figure 5d shows the deposit and consumable rod after the fifth layer deposition. Due to the lack of constraints, a huge flash was formed at the bottom of the consumable feedstock. This part of the material could not be deposited on the surface of the deposit due to the lack of upsetting force, resulting in a certain degree of material waste. It is worth noting that after depositing one layer, the rough top surface of the layer was machined to be flat for the next deposition. The removal of the oxide helps to build up stronger layer interface bonding [10]. Alternating material additive and subtractive processes not only reduced forming efficiency but also increased process costs.

**Figure 6:**
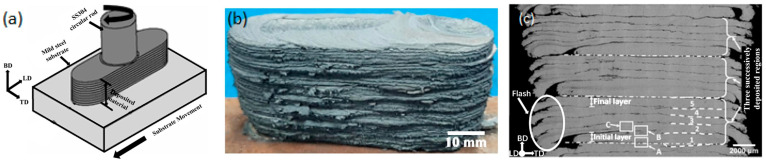
(**a**) Schematic of AFSD, (**b**) deposition of SS304, and (**c**) cross-section. In (**c**), the numbers 1~5 referred to the 1st~5th layers of the deposit. The letters A, B and C with white boxes represented specific regions that have been further characterized in the original research, which we did not discuss in this review [11].

Gotawala et al. [11] prepared SS304 stainless steel deposits using AFSD that consumes print heads, as shown in Figure 6. Unlike the work of Dilip et al. [9], Gotawala et al. [11] did not machine the top surface of each layer in their experiment, but they directly deposited the next layer. Therefore, in addition to the defects mentioned above (flash, poor surface quality, and low forming accuracy), a more obvious interface between layers is also found in Figure 6c, indicating the poor interlayer bonding ability of this process.

### 3.2. Additive Friction Stir Deposition by Non-Consumable Print Head

**Figure 7:**
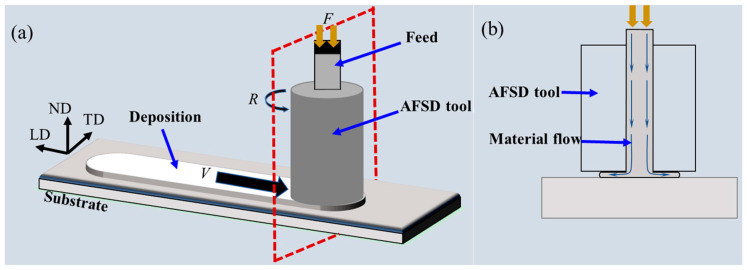
Schematic of AFSD using non-consumable print head showing (**a**) the process and (**b**) material flow [12].

**Figure 8:**
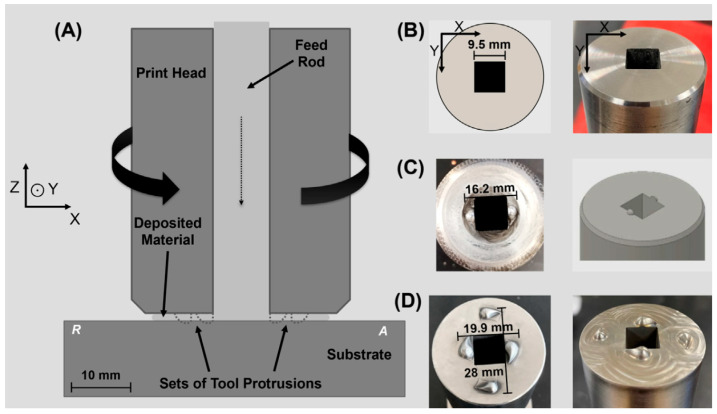
Tool geometry in AFSD using a non-consumable print head. (**A**) A schematic of the print head, feedstock, and substrate and their relative spatial distributions; the dashed line marks the outline of bottom protrusions. R and A represent the retreating side and advancing side, respectively. The photographs of prints with (**B**) 0 protrusion, (**C**) 2 protrusions, and (**D**) 4 protrusions [13].

Compared to additive friction stir deposition by the consumable print head, AFSD using a non-consumable print head has received more attention from researchers due to its better surface quality and interlayer bonding ability. The AM equipment using this type of process has a specially designed hollow print head. Under the action of the feeding device, the feedstock is conveyed to the substrate through the hollow feeding channel inside the print head, and as the print head rotates at high speed, the feedstock is deposited layer by layer on the substrate through the friction stir effect of the print head, as shown in Figure 7. Print heads can be roughly divided into flat-bottom print heads and print heads with protrusions, as shown in Figure 8. The feedstock used in this process can be mainly divided into rods, wires, and powders according to their forms.

#### 3.2.1. Rod as Feedstock

Rods are currently the most common AFSD feedstock in the literature, usually using square cross-section rods. The parameters of this process mainly include the rotation speed of the spindle (ω, rpm), the transverse speed of the print head (*v*, mm/min), the material inserting rate of the feedstock (*f*, mm/min), layer thickness (*h*, mm), and layer width (*l*, mm). From the perspective of equipment principles, these parameters are independently adjustable, but the actual operating process parameters need to approximately satisfy the following equation:

(1) $$f=\frac{v\times l\times h}{d^{2}}\times n$$

where *d* is the side length of the cross-section of the feedstock, mm; n represents the feed constant, *n* ≥ 1.

**Figure 9:**
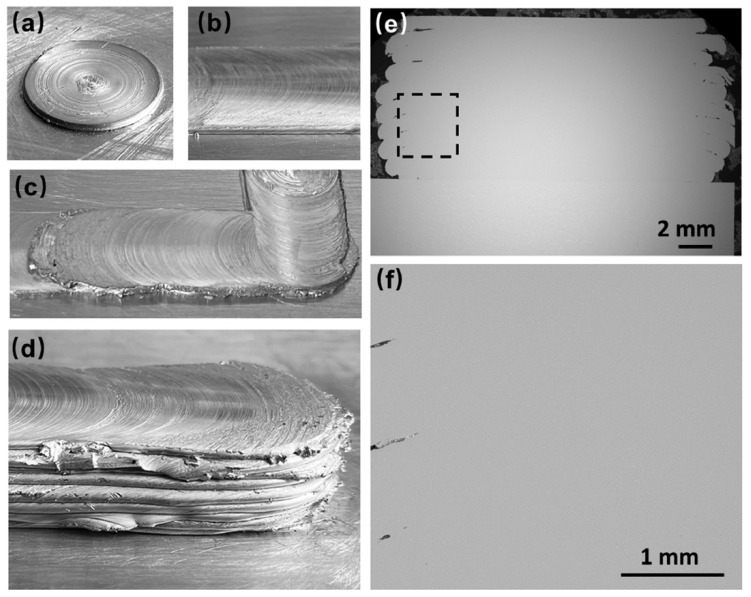
AFSD buildings of 6061 aluminum alloy with the following shapes: (**a**) dot, (**b**) straight line, (**c**) rectangular, and (**d**) multilayer. (**e**) A cross-section of the deposition and (**f**) its high magnification image from the dashed box in (**e**) [14].

Figure 9 shows the 6061 aluminum AFSD deposits using the 6061-T6 rod as the feedstock, from the work of Liu and coworkers [14]. In Figure 9d, it can be seen that the flash phenomenon still existed but was weakened compared to the AFSD that uses the consumable print head. Figure 9e,f show a good interface bonding quality. It is worth mentioning that the area formed by good interfacial bonding was located directly below the print head, while the flash area was outside the print head. In these flash areas, due to the lack of forging and friction stir effect of the print head, there were obvious interfaces between the layers.

**Figure 10:**
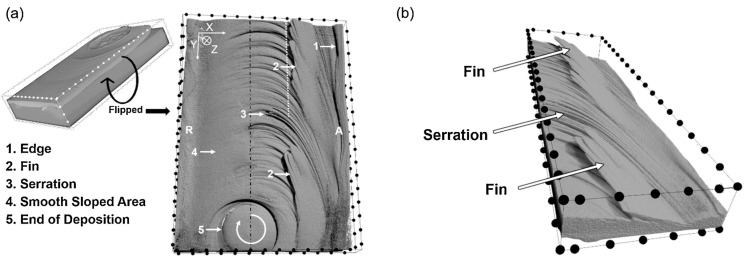
X-ray computed tomography (CT) image of AA2024 deposited onto AA6061 via AFSD. (**a**) Bottom view of the deposited AA2024 material with removed substrate. The distance between adjacent points is 2.5 mm. (**b**) A tilted view emphasizes the fin structures [15].

**Figure 11:**
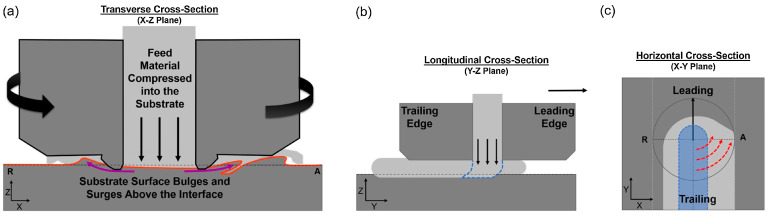
(**a**) Interfacial material flow mechanism on the cross-section along the transverse direction during AFSD. (**b**) Feedstock bending and shearing on the cross-section along the longitudinal direction under the forces created by the in-plane motion of the print head. The dashed line indicates the potential deformation and flow path that plasticized materials might take as they exit the feedstock zone. (**c**) A bottom view of the deposition process on the horizontal cross-section. The deposition track is represented by the light gray area. The initial addition of material via the mechanism shown in (**b**) is represented by the blue area. Curved arrows show the material flow direction after entering the deposition. R and A represent the retreating side and advancing side, respectively [15].

Perry et al. [15,16] investigated the plastic deformation path and the interface formed in AFSD. They used the rodlike AA2024 aluminum alloy as the feedstock and prepared a single-layer deposit on the AA6061 substrate to investigate the interfacial morphology formed during AFSD. The results showed that the material flow induced cross-plane mixing between the printed material and the substrate [15]. The image of X-ray computed tomography (CT) results in Figure 10 show that the interface presented a concave “bowl” structure in the middle. In terms of details, the advancing side formed a “fin” and “serration” structure, while the retreating side was relatively smooth. The authors believed that this was due to the difference in material accumulation within the friction stir zone. As shown in Figure 11, with the rotation of the print head, due to the strong in-plane constraint of excess material on the advancing side, more material accumulated there, causing the AA6061 substrate to move outward and upward, resulting in the accumulated AA2024 moving up and down. This leads to a higher degree of vertical mixing and fin structure formation outside the protrusion area on the advancing side. In contrast, on the retreating side, the in-plane constraint is weaker, the material accumulation is minimal, and 3D structures such as fins will not form.

**Figure 12:**
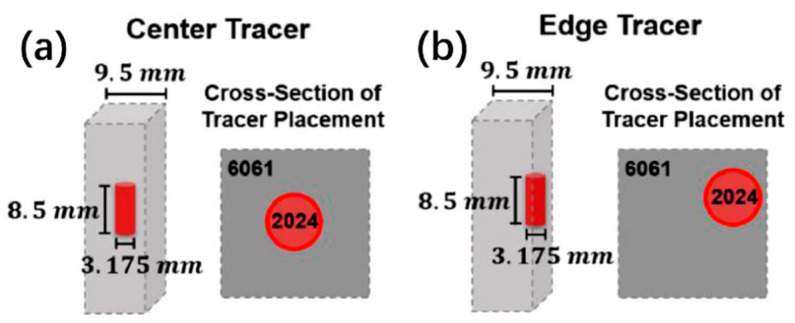
A schematic depicting the dimensions and placement of (**a**) the center and (**b**) the edge tracer within the 6061 feedstock [16].

**Figure 13:**
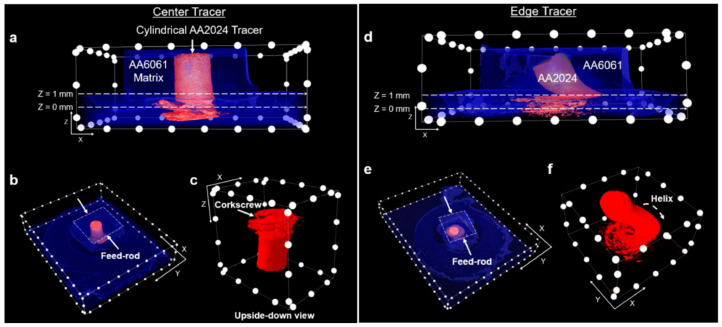
CT results following the initial feeding of the feedstock for the (**a**–**c**) center tracer and (**d**–**f**) edge tracer. The AA2024 tracer is highlighted in red while the encompassing AA6061 matrix is shown in blue or a transparent state. The spacing between each two white dots is 2.5 mm [16].

**Figure 14:**
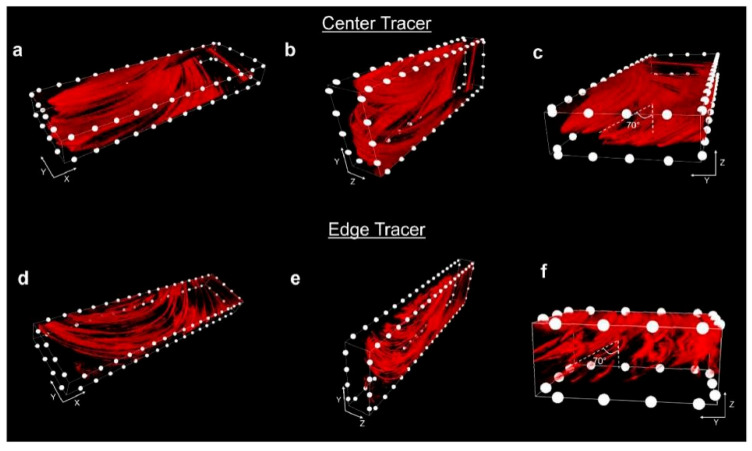
High-resolution CT results following steady-state deposition for (**a**–**c**) center tracers and (**d**–**f**) edge tracers. The spacing between each white dot is 1.25 mm [16].

**Figure 15:**
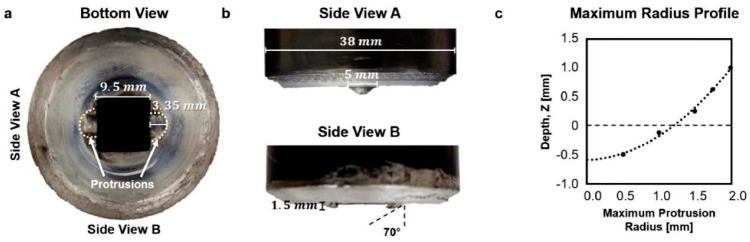
The size and structure of the protrusions: photos from (**a**) bottom and (**b**) side views and (**c**) a graph depicting the protrusion profile. In this plot, the level of the substrate surface prior to printing is set as the reference point, with Z = 0 mm [16].

In Perry and coworkers’ other study [16], they explored the plastic deformation path of AFSD by employing feedstock with different tracers (they plugged the AA2024 tracer in AA6061). They put them at the center and the edge (Figure 12) to show the plastic deformation path at the initial and steady-state feeding stage. The CT results are shown in Figure 13. As we can see, during the initial material feeding, the tracer at the center developed into a corkscrew shape (Figure 13a–c); for the edge tracer, the initial cylinder shape evolved into a helix spanning the entire extent of the feedstock (Figure 13d–f). Compared to above, for both center and edge tracers, the initial millimeter-scale cylinder underwent a striking transformation into curved micro-ribbons, each with a thickness of approximately ten micrometers (Figure 14). Additionally, the micro-ribbons were inclined at about a 70° angle along the *Y*-axis, closely mimicking the contours of the print head’s protrusions (Figure 15).

In addition, Tang et al. [17] also studied the additive process using rodlike 6061-T651 aluminum alloy as the feedstock, but defects such as weak connections and holes were found at the interface. This may be due to the excessive layer thickness (4 mm), which prevented the effective promotion of interlayer material flow by the friction stir effect of the print head.

#### 3.2.2. Powder/Chip as Feedstock

Calvert et al. [18] used an AFSD device (similar to Figure 7) to compare the tensile properties of WE43 Mg alloy AFSD deposits with the powder and rod as feedstock. Fine equiaxed crystal structures were obtained from the deposits using both types of feedstocks (rod and powder), but there were more interfaces between the powders, which increased the number of connection positions between the materials and thus reduced mechanical properties. The ultimate tensile strength (UTS), yield strength (YS), and elongation (EL) of AFSD samples with the rod as feedstock were higher than those with powder.

**Figure 16:**
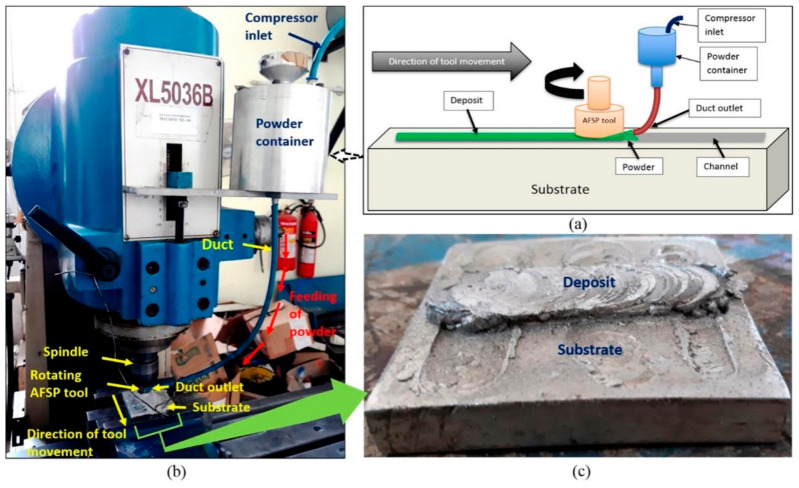
(**a**) An illustration of the schematic and (**b**) the real-life photograph of the AFSP experimental setup along with (**c**) deposition observed on the substrate [19].

Calvert et al. [18] also reported that the solidification of metal powder in a hollow stirring head could hinder further feeding. In response to this issue, Mukhopadhyay and Saha et al. [19,20,21,22,23] designed an external feeding device to achieve AFSD of powder feedstock, as shown in Figure 16. This device used a compressor to transport powder from a container into a duct, which then directed the powder into the preprocessed channel. The metallurgical bonding between the powder and substrate was achieved by the friction stir effect of the print head. This process is also known as additive friction stir processing (AFSP). It is worth mentioning that some researchers have prepared composite materials using the principle of this process, such as the graphite-reinforced 6201 aluminum-based composite material prepared by Liu et al. [24,25,26,27].

**Figure 17:**
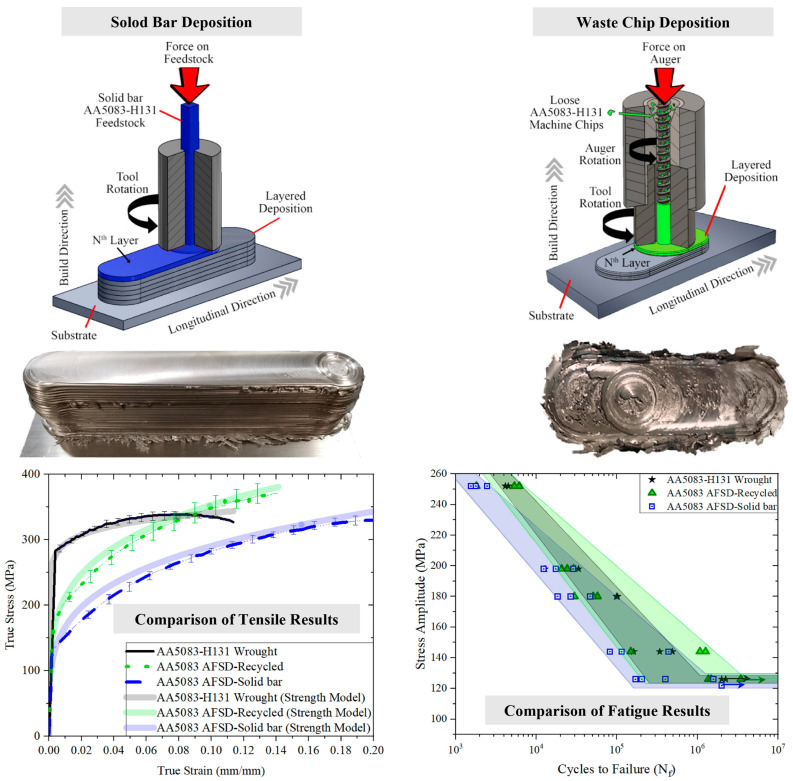
A comprehensive schematic showing the process, morphology of the deposits, tensile curves, and fatigue life [28].

Beck and coworkers [28] successfully conducted the AFSD process on AA5083 directly using machine chip waste as feedstock. From a macro perspective, the appearance of AFSD structures using a rod as feedstock was smoother. Comparison of tensile and fatigue properties between different samples (AFSD using rod, chip, and wrought samples) exhibited that the UTS of both AFSD-rod and AFSD-chip samples increased by 7% on average, the YS reduced by about 37% and 53%, respectively, and EL showed a 14% and 71% increase, respectively. The results of fatigue tests were equivalent for AFSD-rod and AFSD-chip samples, and slightly lower than wrought samples. A comprehensive schematic showing the process, the morphology of the deposits, tensile curves, and fatigue life is in Figure 17. The authors believed that the increase in UTS and fatigue life was attributed to the refinement of grain size, which eliminated the strain-hardening effect thus reducing the YS.

#### 3.2.3. Wire as Feedstock

**Figure 18:**
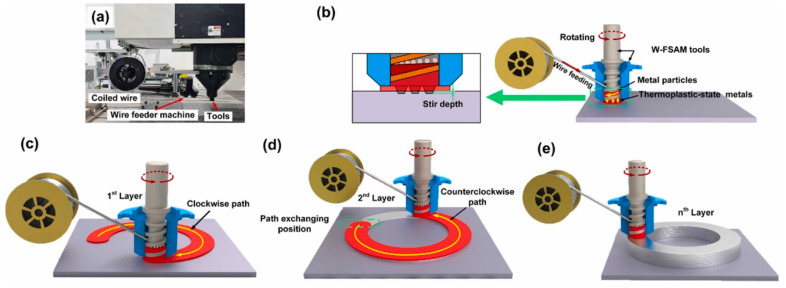
(**a**) Experimental setup for W-FSAM. (**b**) A schematic depicting the process stages: initial stage (dwell stage) of the W-FSAM process, (**c**) deposition of the first layer following a clockwise track, (**d**) deposition of the second layer following a counterclockwise track, and (**e**) repetition of the process until the building attains an intended height [29].

Huang et al. [29,30] designed an AFSD experimental setup using wire as the feedstock (which they called wire-based friction stir additive manufacturing, W-FSAM), and they [29] used this device to manufacture AA4043 aluminum alloy components; Zhang et al. [30] reported that installing the device on a serial robot for AA4043 aluminum alloy additive manufacturing greatly improved the flexibility of AFSD. The process schematic is shown in Figure 18. The main structure of the device includes a stationary sleeve with a wire feeding port and a conveying screw with three protrusions at the bottom. In specific implementations, the screw rotates under the drive of the spindle, shearing the wires into particles, then conveying them downward in the cavity. The compression and shear effects applied by the threads make the metal particles dense and extruded from the end outlet, achieving layer-by-layer deposition of the material under the friction stir effect of the print head. It is worth noting that since the device uses a single screw to simultaneously achieve feeding and printing, the feeding speed and print head rotation speed are not independently adjustable and can only be adjusted together by controlling the spindle speed.

## 4. Microstructure Evolution and Mechanical Properties

As an emerging additive manufacturing technology, AFSD for different alloys (especially light alloys) has been widely studied due to their unique solid-state properties and high deposition efficiency. This section provides a comprehensive look back at the microstructure evolution and mechanical properties of AFSD for different types of alloys. As current scholars have almost exclusively focused on non-consumable print heads; this section will not provide a review of AFSD research on consumable print heads.

**Figure 19:**
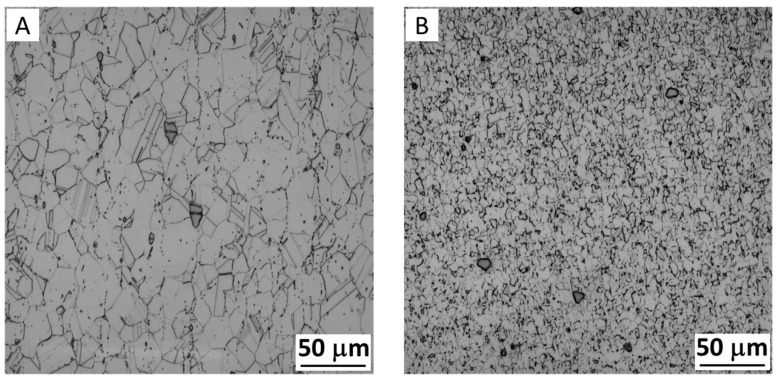
An optical comparison of the microstructure between (**A**) as-received IN625 feedstock and (**B**) IN625 after AFSD [31].

Similar to FSW, the material suffered dynamic recrystallization during AFSD, forming fine equiaxed grains. Figure 19 gives the optical images of an IN625 nickel-based superalloy before and after AFSD, indicating significant grain refinement after AFSD.

### 4.1. Related Research on Aluminum Alloys

One study about AFSD 2195 aluminum alloy was conducted by Li and coworkers [32]. The AFSD 2195 Al–Li alloy demonstrated variations in microstructure and mechanical properties that were influenced by the layer-by-layer process along with the different thermal cycles. In the case of AFSD specimens, the top layer showed superior mechanical properties with a higher YS of 253.7 MPa, a UTS of 409.4 MPa, and an EL of 25.6%, compared to the properties of the bottom layer [32].

**Figure 20:**
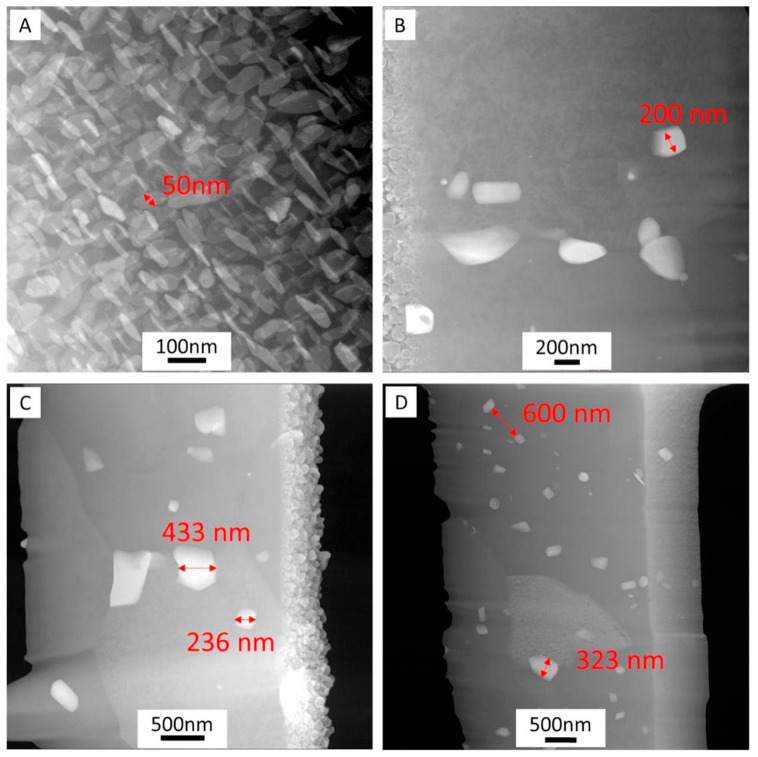
TEM micrographs from the cross-section of (**A**) feedstock material showing θ′ precipitates at the (**B**) bottom, (**C**) middle, and (**D**) top of the building [33].

Rivera et al. [33] conducted AFSD experiments on the AA2219 aluminum alloy and also found the phenomenon of grain refinement. Observation under a scanning transmission electron microscope revealed that after AFSD, the uniformly distributed θ′ precipitates in the feedstock material had disappeared and were replaced by larger and unevenly distributed θ precipitates, as illustrated in Figure 20. The θ phase size distribution gradually increased from the bottom to the top of the deposit, indicating that during AFSD, the peak temperature was high enough to dissolve θ′ phase, and the following cooling rate was low enough to precipitate and grow θ′ phase particles. The closer to the lower layer, the more thermal cycles the material undergoes, and the more obvious the growth of θ phase particles.

**Figure 21:**
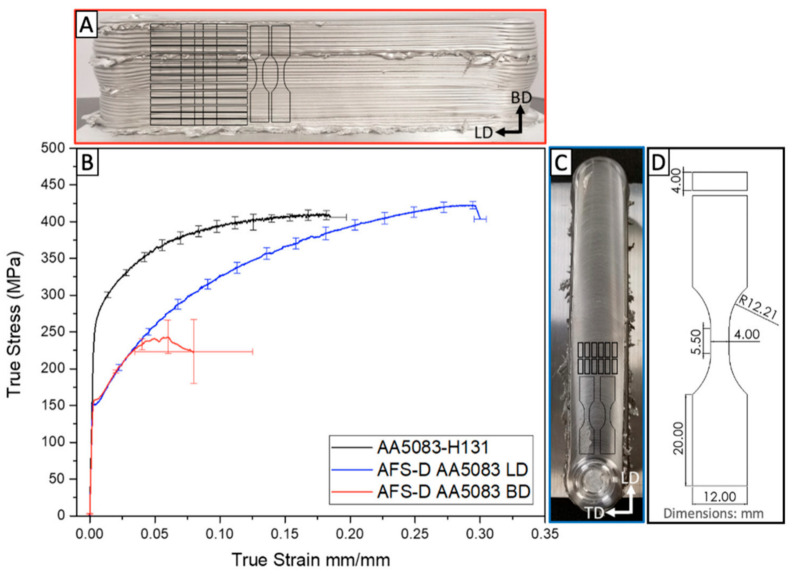
(**A**) A photograph showing the side view of the AA5083 AFSD building. (**B**) The stress–strain curve of the different samples. (**C**) A photograph showing the top view of the AA5083 AFSD deposit. (**D**). The structure and size of the samples in this study [34].

Phillips et al. [34] studied the AFSD process of AA5083 aluminum alloy and compared the longitudinal direction (LD) and building direction (BD) tensile properties of feedstock AA5083-H131 (BM) and AFSD components, as shown in Figure 21 and Table 1. Because of the AFSD process, the BD and LD samples both showed a significant decrease in YS. The thermo-mechanical coupling in AFSD sheared grains through lattice rotation and annihilated dislocations. Thus, the refined grains with almost no dislocations were formed and the work-hardening effects caused by the H131 treatment were almost gone. It is worth noting that in the LD samples, the UTS exceeded that of the AA5083-H131 feedstock by about 20 MPa, and this increase could be attributed to the smaller grain size (3.1 μm). However, the BD sample showed a remarkable decrease in strength–ductility balance. The premature fracture that occurred in the plastic state indicated that there may be defects at the interface between layers due to the layer-by-layer process. It is worth mentioning that in order to prevent the rodlike feedstock from jamming in the feeding channel of the print head during the deposition process, a high-temperature graphite aerosol lubricant was used. However, in EDS analysis, a higher carbon content was found at the failure surface of the BD sample, indicating that the decrease in BD ultimate tensile strength may be caused by the aggregation of graphite in the lubricant between layers.

**Figure 22:**
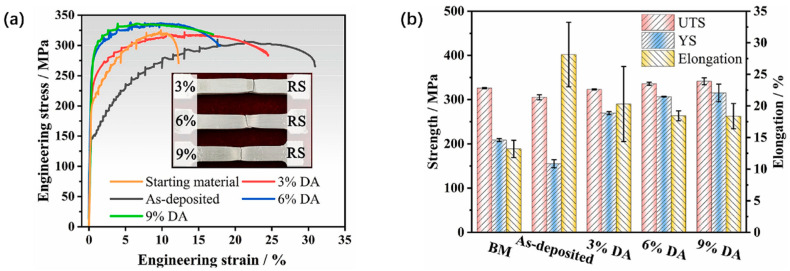
(**a**) Engineering stress–strain curves and (**b**) comparison of YS, UTS, and EL between different samples [35].

Tang et al. [35] applied several certain amounts of compression deformation (3%, 6%, 9%) to the AFSD samples of 5083 aluminum alloy and studied its influence on the microstructure and mechanical properties. The results revealed that the compressed samples demonstrated a remarkable improvement in dislocation generation, accumulation, and their subsequent interactions as compressive plastic deformation was incrementally applied (at 3%, 6%, and 9%). The density of geometrically necessary dislocations (GNDs) rose by a factor ranging from 2 to 5.6, and correspondingly, the subgrain fraction increased from 20.4–34.0% to 43.7–59.8%. Furthermore, all samples showed an increase of at least 73.9% in YS after compressive deformation. The data of the tensile test are shown in Figure 22.

**Figure 23:**
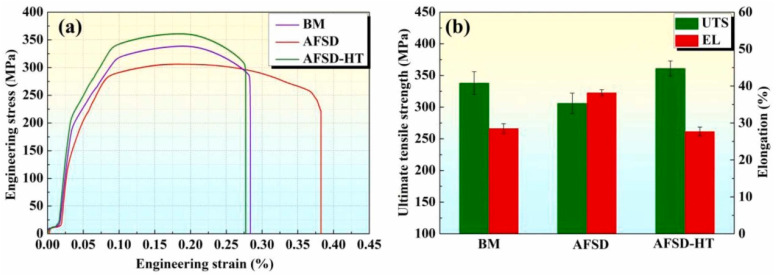
(**a**) Curves of engineering stress–strain curves and (**b**) UTS and EL of BM, AFSD, and AFSD-HT samples [36].

Chen et al. [36] conducted AFSD experiments on the AA6061 aluminum alloy and subjected the deposited components to heat treatment, comparing the mechanical properties of the feedstock, AFSD components, and AFSD components after heat treatment (AFSD-HT). In terms of hardness, because of the dynamic recovery and dissolution of the β″ phase caused by AFSD, the hardness of AFSD components was the lowest (101 HV). After heat treatment, the β″ phase reprecipitated and the grains grew to a certain extent. The hardness of AFSD-HT components was the highest (121 HV). The comparison of tensile properties is shown in Figure 23. Similarly, due to the performance of the β″ phase in AFSD and subsequent heat treatment, the tensile strength and hardness showed a similar trend of change. However, the elongation (EL) of AFSD samples was higher than the AFSD-HT samples. Rutherford et al. [37] believed that this may be due to the lack of strengthening phase β″ among AFSD samples, which reduced the pinning effect of dislocations and enabled a large number of dislocation movements, thereby increasing elongation.

A series of studies were conducted by Tang and coworkers [38,39]. The experimental results on the 6061 aluminum alloy showed that when the layer thickness was set to 4 mm and the transverse speed of the print head was 300 mm/min, the material utilization, deformation, and recrystallization texture fraction at a rotation speed of 400 rpm were 62.5% and 49.2% higher than those at 600 rpm, respectively, and the comprehensive mechanical properties were also better [38]. Furthermore, using 400 rpm for 17-layer deposition, the EBSD analysis indicated a notably higher recrystallization fraction within the interfacial zones compared to the interlayer zones. This higher recrystallization fraction led to an average grain size ranging from 3.4 to 4.2 µm in the interface zones and from 5.3 to 7.8 µm in the interlayer zones. TEM examination revealed that only scattered spherical Al(MnCrFe)Si particles persisted at the newly formed interface, serving as nucleation sites for the subsequent precipitation of Q′ and β′ during the ongoing deposition. The coherent and semicoherent interfaces between the reprecipitated Q′ and β′ and the aluminum matrix contributed to the strengthening of the multilayer deposits [39].

**Figure 24:**
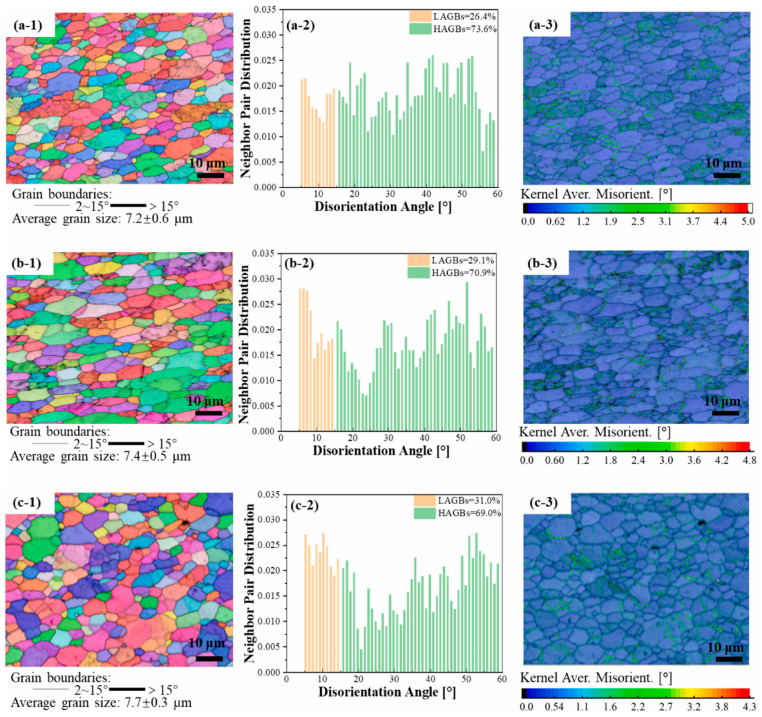
The EBSD results of AA6061 prepared via AFSD under (**a**) 450, (**b**) 500, and (**c**) 550 rpm. The EBSD map, misorientation angle histograms, and KAM are represented by suffixes (**1**), (**2**), and (**3**) [40].

**Figure 25:**
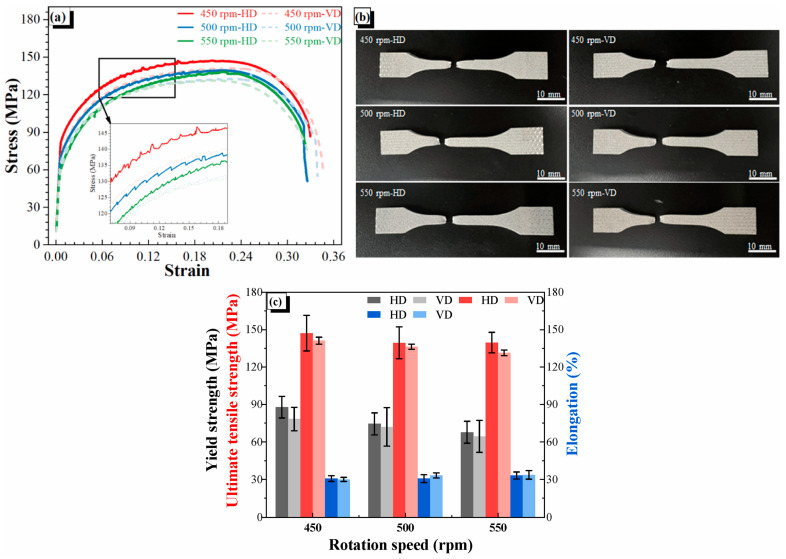
(**a**) The engineering stress–strain plots of different samples. (**b**) The tensile sample after the test showed the fracture behavior. (**c**) A bar chart summarizing the mechanical properties of different samples. HD and VD represent the horizontal and vertical direction, respectively [40].

Qiao et al. [40,41] studied the influence of process parameters (rotation speed and layer thickness) on the microstructure and mechanical properties of AA6061. As the rotation speed increased (450, 500, and 550 rpm), the grain size showed an increasing trend (7.2 ± 0.6 μm, 7.4 ± 0.5 μm, and 7.7 ± 0.3 μm, respectively), and the kernel average misorientation showed a decreasing trend (5.0, 4.8, and 4.3, respectively), as shown in Figure 24 [40]. The finer grains at 450 rpm were attributed to lower heat input and plastic flow degree [42]. In terms of mechanical properties, the results are summarized in Figure 25; as we can see, strength (YS and UTS) showed a decreasing trend, but the EL showed a slightly increasing trend [40]. For layer thickness, as it increased (0.5, 1.0, and 1.5 mm), the mechanical properties exhibited a similar trend to the decrease in rotation speed [41].

The study of residual stress on AA6061 prepared by AFSD was conducted by several scholars [43,44]. Regarding neutron diffraction, they reported that the residual stresses in both starting and center zones were tensile stresses, with values of 5~91 MPa and 5~76 MPa for LD and TD, respectively. And the ending location exhibited compressive residual stresses of −127~−46 MPa. Due to the minimal number of thermal cycles experienced, the maximum residual stresses (~−127 MPa) were observed at the top layer [43]. Another study using XRD suggested that residual stresses in the substrate were dominated by compressive stress [44]. These values are slightly higher than the 6061 parts manufactured by wire-arc additive manufacturing [45]. Although excessive residual stress can reduce fatigue performance [46,47,48,49,50], considering that AFSD components have almost no voids, perhaps the comparison of fatigue life will show different results.

**Figure 26:**
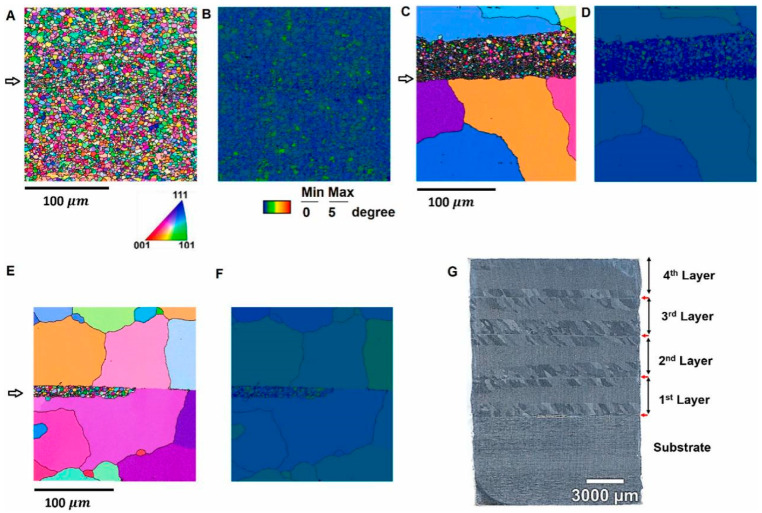
EBSD and optical microscopy images of theAA7050 deposits. (**A**) IPF and (**B**) GAM from the interface zone between the 2nd and 3rd layers. (**C**) IPF and (**D**) GAM from the interface zones between the 2nd and 3rd layers after solution treatment. (**E**) IPF and (**F**) GAM from the same zone after T74 tempering. (**G**) Optical microscopy image of the whole deposit after heat treatment from the cross-section [51].

**Figure 27:**
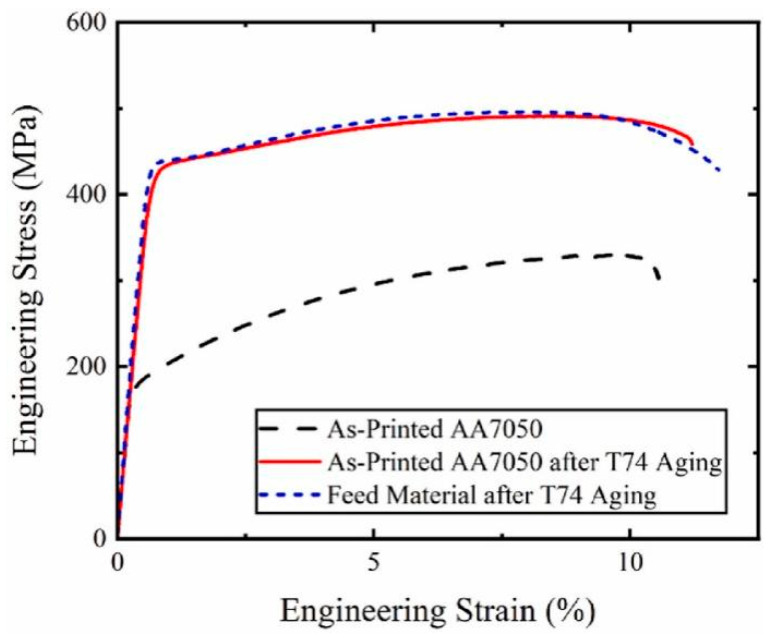
Engineering stress–strain curves of different AA7075 samples (as-printed AA7050 and as-printed AA7050 after T75 aging and feed material after T74 aging) [51].

Hahn et al. [51] conducted an AFSD experiment on the AA7050 aluminum alloy, subjected the AFSD samples to T74 heat treatment, and then characterized their microstructures and tested their mechanical properties. The research found that the as-deposited AA7050 weakened due to the excessive growth of precipitated phases, and its strength was enhanced by T74 tempering. Heat treatment induced abnormal grain growth near the layer interface, which appeared to have the least impact on in-plane tensile properties. The microstructures and stress–strain curves of the tensile results of each sample are illustrated in Figure 26 and Figure 27. It confirmed that after T74 heat treatment, the AFSD sample exhibits forging-like tensile properties.

Sun and coworkers [52] achieved additive manufacturing of TiB_2_/7050 aluminum-based composite materials via AFSD. Following the AFSD process, the TiB_2_ particles within the composite were found to be evenly dispersed. The AFSD sample exhibited a reduced average grain size of 2.17 μm and a lower average aspect ratio of 1.78, in contrast to the characteristics of the feedstock.

**Figure 28:**
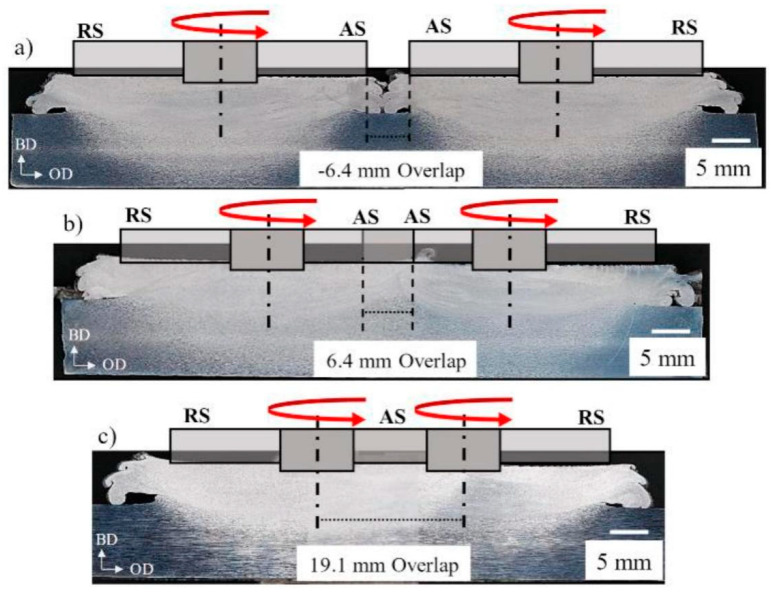
(**a**–**c**) Optical microscope images from the cross-section of the AFSD deposits; the schematics represent the profile of the print head [53].

**Figure 29:**
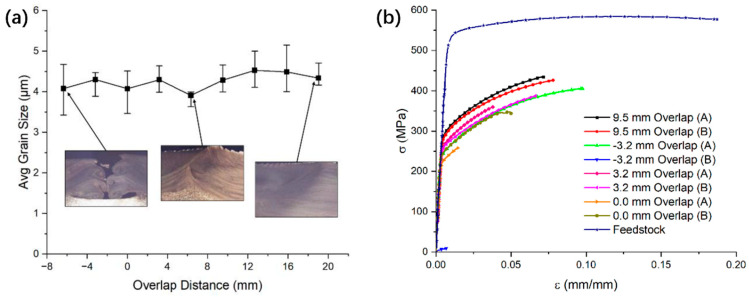
(**a**) A plot depicting the relationship between grain size and the overlap distance within the AFSD samples. (**b**) Quasi-static test data for AFSD samples with varying overlap distance, compared to the feedstock [53].

For the 7075 aluminum alloy, Cahalan et al. [53] investigated the influence of deposition pass overlap width on the deposit produced by the AFSD process. The schematics of three representative overlap buildings with the location of the print head are shown in Figure 28. The grain size and tensile test results of AFSD overlap samples are shown in Figure 29a,b. Obviously, as the amount of overlapping increased, the ductility of the material was also enhanced.

### 4.2. Related Research on Magnesium Alloys

Magnesium and its alloys continue to play an important role in applications requiring lightweight materials and advanced devices. The annual rise in the use of magnesium (Mg) reflects an increasing market demand for Mg-based alloys [54]. A series of studies on AZ31 magnesium alloys were conducted by researchers. Robinson et al. [55] found that the AFSD process notably enhanced the texture intensity and the fiber texture in comparison to the feedstock, particularly for the X–Z and Y–Z planes. However, the X–Y plane maintained a texture intensity akin to the feedstock, but without fiber texture [55]. Previous research on texture development during FSW [56,57,58,59,60] found strong texture formation in the stir zone for Mg alloys lacking rare earth elements, which was heavily influenced by factors such as geometry, plunge depth, and rotation speed of the tool, and specific location within the welding zone. These FSW studies on texture concurred with the findings of Robinson’s study on the bulk AFSD of AZ31B. They also conducted the quasi-static tensile test on AFSD AZ31B and compared the results with the extruded feedstock [55], rolled plate [61], O temper [62], and additive friction stir layer welding (AFSLW) [62]. From their results, it is evident that the AFSD AZ31 samples possessed a microstructure and mechanical properties similar to forging ones.

**Figure 30:**
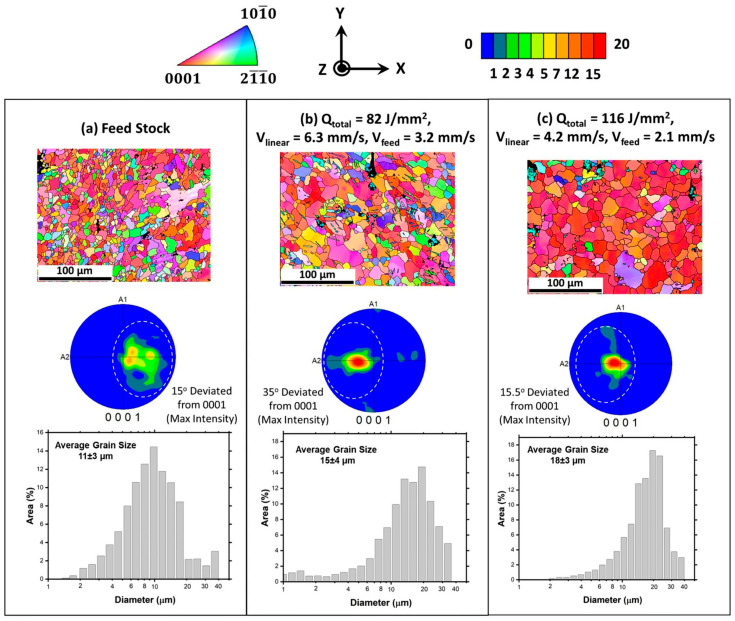
The EBSD results containing IPF and PF, and the distributions of grain size from (**a**) feedstock, (**b**) 82 J/mm^2^, and (**c**) 116 J/mm^2^ samples. The 82 J/mm^2^ sample was associated with a process parameter of 400 rpm and 4.2 mm/s. The 116 J/mm^2^ sample was associated with a process parameter of 400 rpm and 6.3 mm/s [63].

**Figure 31:**
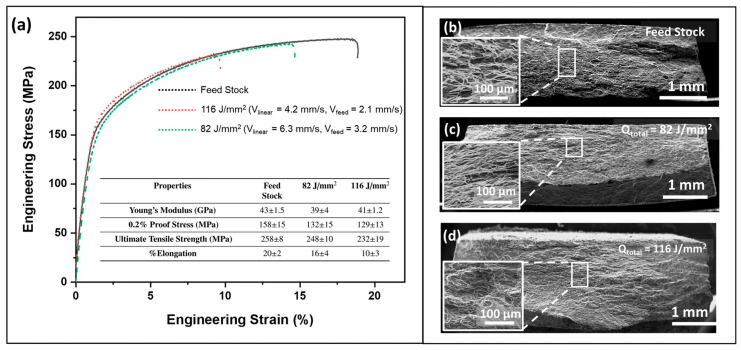
(**a**) Engineering stress–strain curves of different samples. The fracture images of (**b**) feedstock, (**c**) 82 J/mm^2,^ and (**d**) 116 J/mm^2^ were obtained by SEM. The 82 J/mm^2^ sample was associated with a process parameter of 400 rpm and 4.2 mm/s. The 116 J/mm^2^ sample was associated with a process parameter of 400 rpm and 6.3 mm/s [63].

Joshi et al. [63] prepared AZ31B magnesium alloy samples under different parameters (400 rpm—6.3 mm/min; 400 rpm—4.2 mm/min) with a density of ≥99.4% using AFSD and characterized their microstructures and tested their mechanical properties. Compared with the feedstock, the samples produced by AFSD experienced an evolution of (0001) texture at the surface, with a slight increase in grain size. The fraction of Mg_17_Al_12_ in the AFSD samples decreased; the 0.2% proof stress of the AFSD samples decreased by about 30 MPa, and the tensile strength decreased by 10–30 MPa; and the elongation of AFSD samples was 4–10% lower than that of feedstock. Comparing the two samples under 6.3 mm/min and 4.2 mm/min, as the transverse speed decreased, the average grain size exhibited a slight increase from 15 ± 4 μm to 18 ± 3 μm, and the mechanical properties (YS, UTS, and EL) showed a reducing trend. The detailed results are illustrated in Figure 30 and Figure 31.

**Figure 32:**
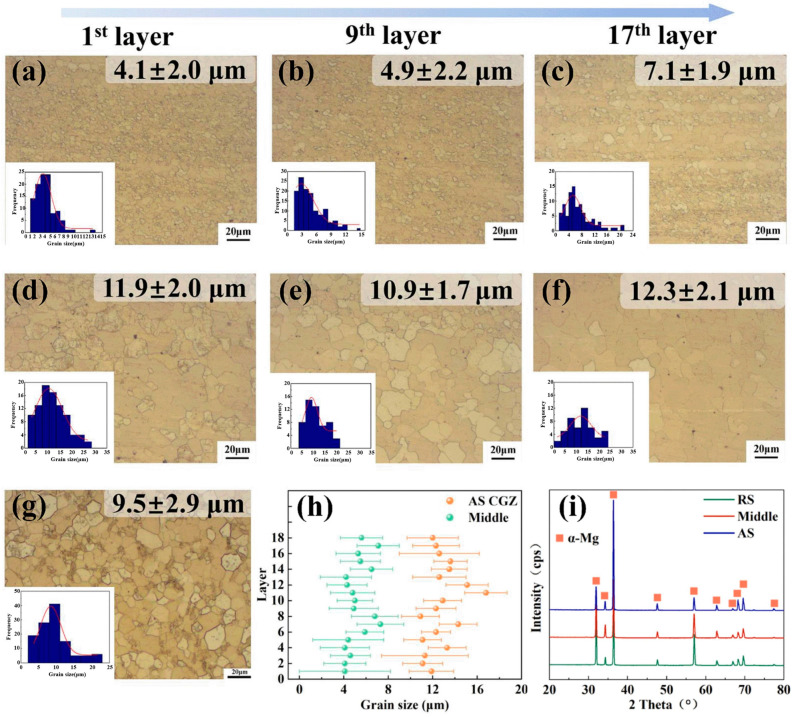
OM images taken of the (**a**–**c**) middle zones and (**d**–**f**) coarse-grain zones from the AS and (**g**) feedstock. Additionally, (**h**) layer-by-layer grain size statistics were compiled, and (**i**) XRD results were obtained [64].

**Figure 33:**
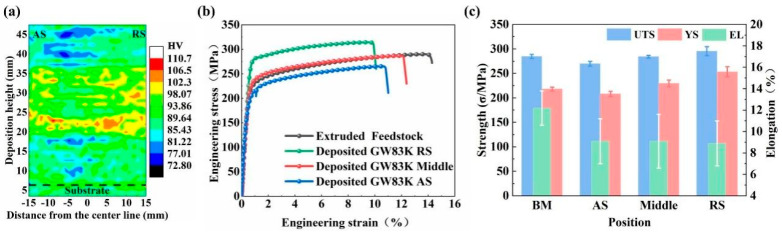
(**a**) Hardness distribution, (**b**) curves of engineering stress–strain, and (**c**) a summary of the mechanical properties of the AFSD deposits [64].

Luo et al. [64] successfully prepared multilayer unidirectional pass deposits of high-performance Mg-8Gd-3Y-0.5Zr alloys via force-controlled AFSD (they called it friction extrusion additive manufacturing, FEAM). Their results indicated that the grain size within the final deposits was not uniformly distributed across the width. A coarse-grain zone, characterized by an average grain size of 12.8 ± 1.5 μm, was observed on the advancing side (AS) of AFSD builds (Figure 32). This coarser grain structure diminished the fine grain strengthening effect, resulting in an average UTS of only 269.9 MPa in that zone. In contrast, the UTS for the middle and RS zones were higher, reaching up to 284.4 MPa and 295.4 MPa, respectively (Figure 33), which was attributed to the significant grain refinement that occurred during the deposition process.

**Figure 34:**
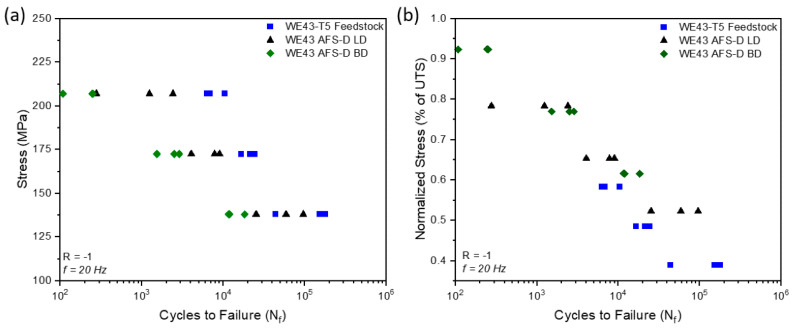
(**a**) The S-N plot and (**b**) the S-N plot normalized by UTS generated for the load-controlled fatigue test of the 68-layer WE43 deposits, examining the BD, LD, and WE43 T-5 feedstock, across three different stress levels [65].

Williams and colleagues [65] investigated the influence of process parameters on the microstructures and mechanical properties of WE43 magnesium alloys prepared by AFSD. In particular, studies on process parameters were conducted through several four-layer builds to determine the appropriate process parameter window for WE43 deposition with the 68-layer structure. The parameter study identified a set of acceptable parameters that result in components with minimal surface defects and high interlayer bonding quality. The microstructure, tensile properties, and fatigue life were characterized and subsequently compared to commercial forged materials. This comparative analysis was conducted to elucidate the relationship between the process, structure, and performance of materials produced via AFSD. This research indicated that the 68-layer WE43 deposits presented fine and uniform microstructures and texture changes relative to the forged material. However, compared to the forging WE43, a decline in hardness and tensile properties was noted in AFSD WE43 samples. In addition, fatigue specimens extracted from large, deposited blocks showed a decrease in lifespan under low-cycle conditions, but their performance under high-cycle conditions was comparable to that of forgings (Figure 34). The results of this research demonstrated the capability of AFSD in manufacturing load-bearing structural components from WE43 alloys.

McClelland et al. [66] compared the mechanical properties of WE43 in different conditions, as reported in the literature (Table 2). The material produced via AFSD exhibited greater YS and UTS than both the underaged WE43 and the WE43-T6 tempered versions, while also maintaining a similar level of EL.

### 4.3. Related Research on Other Alloys

**Figure 35:**
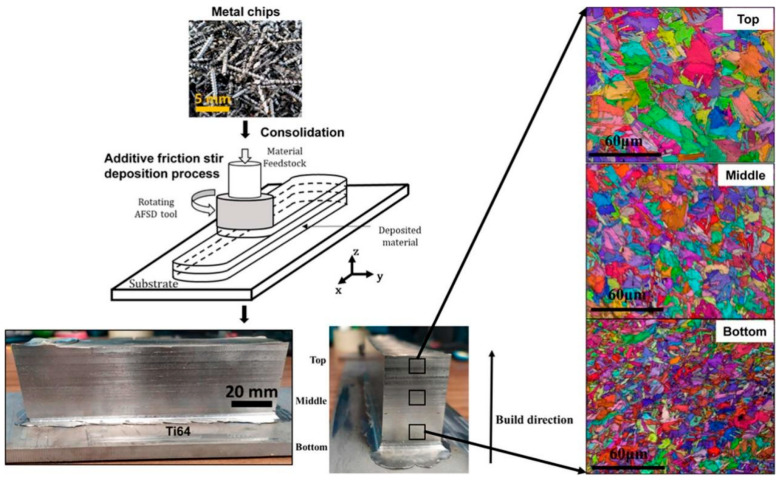
A schematic of AFSD using recycled Ti64 chips and the EBSD map from the top, middle, and bottom of the deposits [68].

Agrawal et al. [68] used recycled metal chips to prepare feedstock through consolidating, and the AFSD of Ti-6Al-4V (Ti64) was successfully achieved through such feedstock; the resulting deposits demonstrated superior tensile properties compared to those produced by other AM processes. Figure 35 shows the schematic of AFSD using recycled Ti64 chips and the EBSD map from the top, middle, and bottom of the deposits. Figure 35 indicates a spatial gradient in the microstructure across the deposit; specifically, specimens taken from the zones close to the substrate (the bottom region) exhibited smaller prior β phase grains and smaller sizes of lath. This difference was attributed to the more rapid cooling rate that the deposited material experienced in this zone. The results of the tensile test revealed that the size of the prior β grain and lath determined the mechanical properties. The as-deposited samples showed an EL of 7 ± 1%, a YS of 1050 ± 25 MPa, and a UTS of 1140 ± 20 MPa.

**Figure 36:**
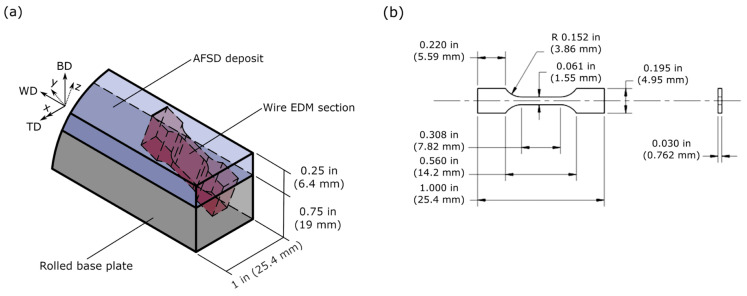
(**a**) A schematic of the AFSD builds on a rolled substrate showing the sampling location of the tensile samples. The sample forms a 20° inclination with BD. (**b**) The structure and size of the samples [69].

**Figure 37:**
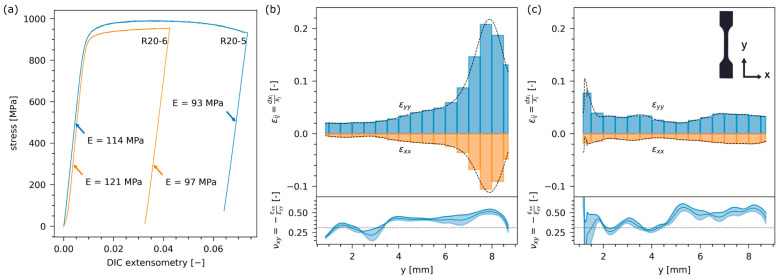
DIC derived stress–strain curves are presented in (**a**), along with the average final strain components (*ϵ*_*ij*_) calculated across the gauge width (– –) and within the region analyzed by PXRD (colored boxes) for (**b**) sample R20-5 and (**c**) sample R20-6. Below these plots, Poisson’s ratio (*ν*_*xy*_) for the two strain components is graphed, compared with the standard bulk elastic value (…). The shaded zone represents the standard deviation observed in the transverse (x) direction [69].

**Figure 38:**
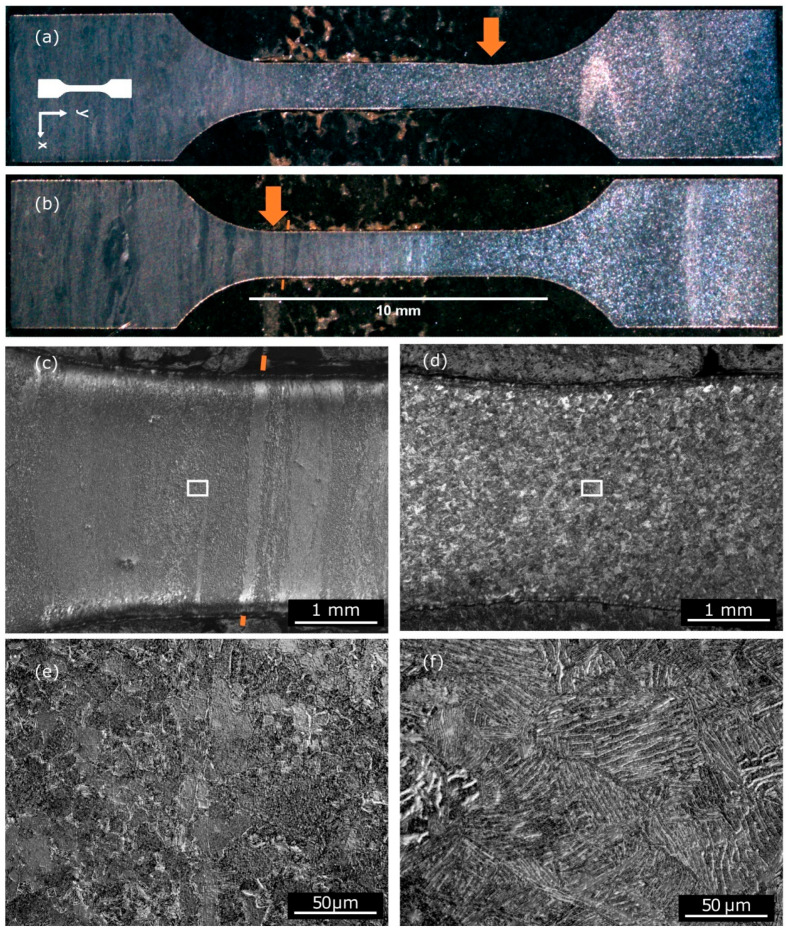
Stereomicroscope images showing the metallographic preparation of the two Ti64 tensile samples: (**a**) sample R20-5 and (**b**) sample R20-6. The arrows in the images represent the areas of strain localization (or necking), which occurred due to uniaxial loading. Images of crossed–polarized bright-field optical microscopy at maximum strain localization: at 5× magnification for (**c**) sample R20-6 and (**d**) sample R20-5; and at 100× magnification for (**e**) sample R20-6 and (**f**) sample R20-5. The orange marker annotation highlights the band in (**b**,**c**), while the boxes in (**c**,**d**) indicate the areas magnified in subsequent figures [69].

Metz et al. [69] studied Ti64 deposited by AFSD onto a rolled Ti64 substrate. In the tensile test with digital image correlation (DIC), the tensile specimens extracted from both the substrate and AFSD regions (Figure 36, two samples were labeled as R20-5 and R20-6, respectively, where the integer suffix indicated the height relative to the substrate) showed different stress–strain responses (Figure 37a). These differences were rationalized and explained by the microstructure gradient obtained by an optical microscope (Figure 38) and spatially resolved laboratory X-ray powder diffraction (PXRD, Figure 37b,c). The AFSD region exhibited a unique “basket weave” shape α-Ti in the prior β grains of 25–50 μm. The substrate showed a globular *α* + *β* microstructure which is typical for material that has been rolled and annealed. In one of the samples, plastic strain concentration was noted within the region processed by AFSD, and EBSD results showed that the directions of *α*-Ti were subjected to preferential loading. In another sample, strain concentration was observed within the rolled substrate, but the region of texture was more limited in size than the zone investigated by PXRD. The manipulation of the prior *β* microstructure through the precise control of deposition temperature, shear rate, and axial stress will be essential for achieving real-time volumetric control and optimizing properties when AFSD is applied to titanium alloys.

Agrawal et al. [70] delved into the parameters’ optimization, microstructure evolution, and recrystallization associated with the deposition of SS316 stainless steel, which has low stacking fault energy, via the AFSD. The deposited material exhibited an equiaxed ultrafine-grained microstructure, characterized by an average grain size of approximately 5.0 ± 0.5 μm. The high-temperature shear deformation incurred during processing triggered the recovery mechanism. Due to discontinuous dynamic recrystallization occurring during the deposition process, the deposited SS316 exhibited a distinctive necklace-like microstructure. The recrystallization kinetics of SS316 throughout the AFSD process were quantified using the Johnson–Mehl–Avarami–Kolmogorov model (JMAK). The variable recrystallization kinetics were attributed to thermal cycles caused by the friction stir effect during the AFSD process. Fluctuations in temperature, strain, and strain rate, influenced by process parameters during AFSD, induced changes in the microstructure and tool wear. The synergetic improvement of strength and ductility, coupled with a persistent work-hardening effect in the SS316 deposits was believed to stem from deformation-induced transformations and the occurrence of twinning. This led to the formation of a gradient in twinning and martensitic phases following deformation.

**Figure 39:**
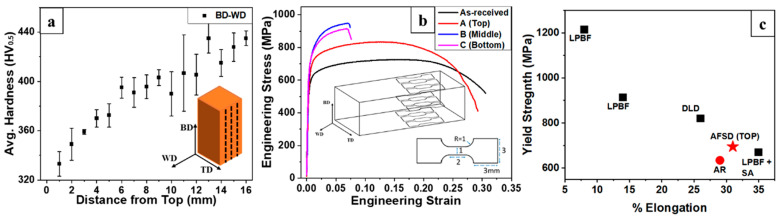
A summary of mechanical properties. (**a**) The relationship between microhardness and the distance to the top surface in the DSS2507 deposit. (**b**) Engineering stress–strain curves of different DSS2507 samples; the illustration shows the structure and size of the samples. (**c**) A comparison of the YS and EL of DSS2507 samples produced by AFSD and other AM methods [71,72,73,74].

Gor et al. [71] successfully prepared the deposits of DSS2507 duplex stainless steel via AFSD and reported the microstructure evolution and mechanical properties. The results showed that AFSD processing remarkably affected the banded microstructure of the feedstock. The austenite grains displayed a fine, uniform, and equiaxed structure; in contrast, the ferrite grains were somewhat larger and displayed an elongated morphology. According to observations of the microstructures, the authors inferred that the discontinuous dynamic recrystallization (DDRX) was a potential mechanism for the microstructure evolution of austenite, whereas in the ferrite, the potential mechanism was thought to be continuous dynamic recrystallization (CDRX). They also found that the σ phase precipitated during the AFSD process, which was attributed to the multiple thermo-mechanical cycles introduced by the AFSD process; this precipitation in turn caused significant variations of mechanical properties along BD. Comparing the DSS2507 feedstock with other AM DSS2507 parts, the top of the AFSD deposits with a non-significant percentage of the σ phase displayed a synergetic improvement of YS and EL, and the results of the mechanical property tests are shown in Figure 39.

## 5. Opportunities and Challenges

Although AFSD has unparalleled advantages in manufacturing large or lightweight alloy parts, the forming quality, including rough surface and flash, has always been an obvious problem. In terms of microstructures and properties, there remains some ambiguity in the relationship between them and the process parameters. All of these relationships require further research.

### 5.1. Improvement of Molding Quality and Spatial Resolution

At present, almost all components manufactured by AFSD require subsequent subtractive processing [75]. Compared with other types of metal additive manufacturing, AFSD has more serious problems with rough surfaces and flash, which requires cutting more materials during subtractive processing, thus exacerbating material waste and increasing process costs.

**Figure 40:**
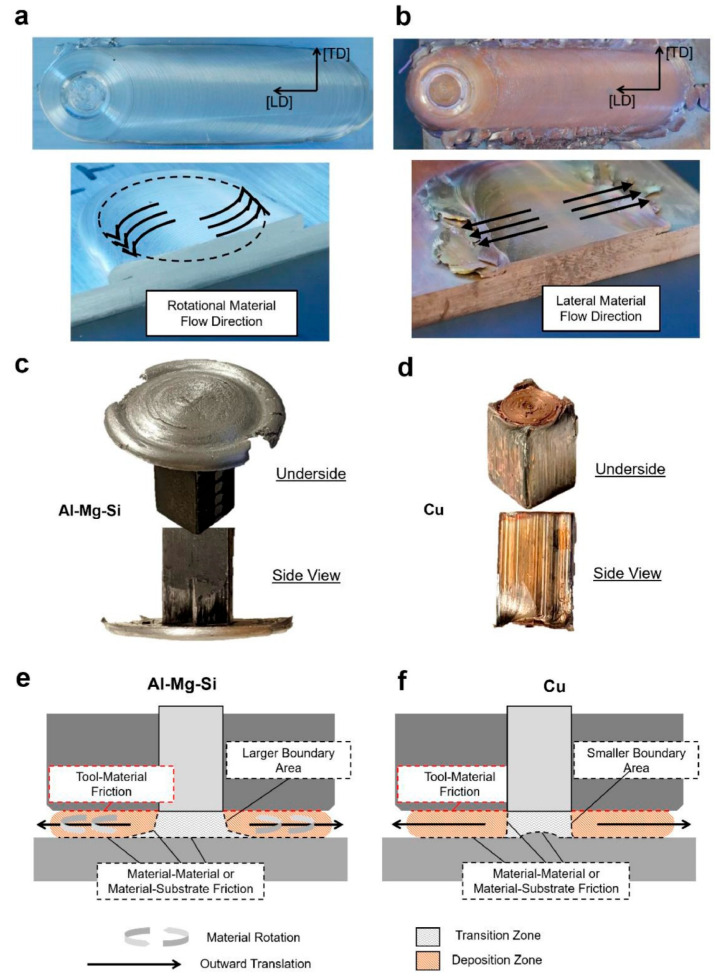
A comparison of the deformation patterns in AFSD for Al-Mg-Si and Cu alloys. Photographs of the deposits, including the top and cross-sectional views of the (**a**) Al-Mg-Si and (**b**) Cu alloy. The photographs of the end of the remaining feedstock after deposition of (**c**) Al-Mg-Si and (**d**) Cu. Schematics show the mechanism of flow and deformation of (**e**) Al-Mg-Si and (**f**) Cu [78].

The rough surface along the building direction is mainly caused by flash, and in addition to process parameters, the severity of flash has a strong dependence on the properties of the material itself, especially the adhesion coefficient. In FSW, the adhesion coefficient may vary locally (decreasing with increasing surface contact velocity) [76,77]. In order for materials to adhere, a higher coefficient of friction is required to provide sufficient shear stress to drive material flow. Griffiths et al. [78] compared the AFSD processes of the 110 copper alloy (Cu) and the 6061 aluminum alloy (Al-Mg-Si) and analyzed the different flash behaviors of the two deposits. It has been reported that aluminum–steel has a higher friction coefficient than copper–steel [79]; hence, it was observed that in the case of the 6061 aluminum alloy, there was significant rotational motion in the AFSD zone, with excess feeding materials leaving the area directly below the print head, resulting in the formation of a circular structure (as shown in Figure 40a,c,e). In contrast, the motion of rotation was not obvious in Cu’s AFSD zone, so, under the effect of conveying new feedstock, the previously deposited materials flowed laterally. Therefore, the spatter left the deposition zone in the form of flat plates along the lateral direction (as shown in Figure 40b,d,f). Due to the additional shear stress generated by the rotational material flow across most of the deposition area, Al-Mg-Si underwent more severe deformation than Cu.

**Figure 41:**
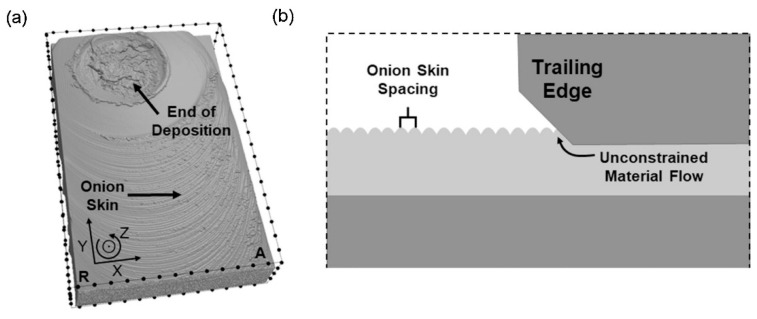
The deposition of AA2024 onto the AA6061 substrate. (**a**) The top view acquired by CT. The distance between adjacent points is 2.5 mm. (**b**) A schematic describing the mechanism of the formation of the “onion skin” structure [15].

As shown in Figure 9c,d and Figure 41a, the upper surface of the deposited layer was relatively rough, exhibiting the characteristic “onion skin” structure [15]; a similar structure has also been observed in FSW [80,81,82,83]. The “onion skin” structure is produced through the interaction at the boundary where the edge of the bottom surface of the print head meets the top surface of the newly deposited material with each revolution. As the print head rotates and moves forward, the trailing edge of the print head applies a kind of milling-like effect to the surface of the deposited material. As a result, marks similar to “onion skin” were formed [15]. The schematic describing this mechanism is shown in Figure 41b.

The spatial resolution of AFSD is primarily reflected in the width of each print layer, which depends on the geometric parameters of the print head—especially the bottom diameter. Therefore, the most direct method to improve spatial resolution is to use a print head with a smaller diameter. However, as the size of the print head decreases, the heat generated by the friction stir effect may become insufficient [76]. And because of the high forging force during the AFSD process, this process may not be suitable for forming thin-walled structures, which means that we cannot reduce the bottom diameter of the print head without limitation. To enhance spatial resolution while maintaining printing efficiency, introducing an external heat source (such as inductive preheating, laser heating, etc.) and a specially designed support structure would be an ideal solution.

Starting from the principle of flash and surface “onion skin” formation, the methods to improve the above problems lie in optimizing process parameters and improving the geometric shape of the print head. The feeding speed during printing is reasonably set to reduce excessive material supply, thereby preventing excessive material from leaving the deposition zone from the edge of the print head; the relationship between the rotation speed and transverse speed of the print head is properly matched, so that each “onion skin” mark has a certain overlap, thereby reducing the milling marks of the print head. Print heads with new structures are developed, such as applying constraints on the lateral flow of materials through geometric structures to reduce the formation of flash, and smoothly transitioning the edges of the print head to reduce milling effects.

### 5.2. Control and Improvement of Microstructure and Performance

Although the AFSD process can usually form fine equiaxed grains, grain size and morphology may be influenced by many factors. In order to obtain an ideal fine equiaxed microstructure, it is necessary to establish a relationship between microstructure evolution and process parameters for different materials. In terms of achieving uniform and high mechanical properties, it is important to carefully regulate the microstructures at the sub-micron level, such as precipitates, intermetallic compounds, and even nanoscale microstructures, including grain boundaries and dislocations [5]. All the above content about microstructure evolution requires further research.

Grain refinement during AFSD may result in fine grain strengthening, but, for some alloys, the AFSD process may weaken the precipitation hardening effect; one example is aluminum alloys. The dissolution of θ′ precipitates in AA2219 [33] and the dissolution of β and β′ precipitates in AA6061 [84] typically lead to a decrease in the tensile properties of AFSD samples. The lack of precipitate leads to a decrease in the hardness of AFSD components, especially those made from feedstock under optimal heat treatment or forging conditions.

Because of the continuous interaction with the deposited materials, the print head used in AFSD undoubtedly faces wear issues, especially when manufacturing hard metals such as titanium. Although there is no directly related research on the wear issue of AFSD print heads, the research on FSW or friction stir processing (FSP) tools also has great guiding significance. Amirov et al. [85,86] reported that, when using Ni-based tools for FSP on pure titanium, as the wear of tools intensified, voids became more obvious in the stirring zones, and the tensile properties (YS, UTS, and EL) showed a decreased trend. A similar trend in strength was also observed in 2219 aluminum alloys [87]. Amirov et al. [85,86] also reported that water-cooling tools could slow down their wear rate; the same methods and conclusions may be worth learning from by AFSD researchers.

Regarding the appeal issue, in addition to conducting a large number of relevant experimental studies, relevant computer simulation research is also particularly important. But the simulation studies on AFSD are insufficient now. However, due to the similarity of the process, researchers can find suitable simulation models for AFSD from the computer simulation studies of FSW/FSP. Apart from mechanical properties, other properties, such as corrosion and biocompatibility should be considered to expand the application areas of AFSD. A series of research studies on Mg-Li alloys [88,89,90], Mg-RE alloys [91], and Al alloys [92] prepared by FSP were conducted by Zhou, Zhu, and coworkers, and the promising results of corrosion resistance and biocompatibility were reported. Given the similar principles of FSP and AFSD, the methods and results of the above research have huge significance for AFSD.

### 5.3. Optimization of Process Parameters

At present, the most common process parameter optimization is still based on experiments and involves setting a series of process parameters for AFSD and comparing the macroscopic morphology and properties of deposits under different parameters. According to current information, the common control strategy for AFSD devices is displacement control. In FSW/P, there are usually two control modes, force control and displacement control [93], while in actual industrial production, force control is generally considered to have greater advantages than displacement control. Yang et al. [17,35,38,39,64,94] developed AFSD equipment based on force control, which shows great potential in terms of parameter optimization such as parameter tuning and real-time adjustments.

Recent research on artificial intelligence has dispelled the fog surrounding the parameter optimization of AFSD. Although there is no directly related research, some models and methods [95,96] from other fields are still worth studying further for researchers in AFSD.

## 6. Conclusions

This article provides a critical review of recent AFSD research, with a focus on macroscopic formability, material flow, mechanical properties, and microstructure evolution. The key conclusions are summarized in Table 3 for quick reference.

The solid-state AFSD, as a novel AM method, has enormous application prospects in aerospace, automotive, marine, defense, and equipment fields because of its unique advantages. Scholars have conducted relevant research on the AFSD of different materials such as aluminum alloys, magnesium alloys, stainless steel, nickel-based alloys, etc., confirming their feasibility, and focusing on the microstructure evolution and mechanical properties. However, research on AFSD is still in its infancy, and some mechanisms, especially recrystallization, dissolution–precipitation, material flow, and other behaviors during the process, require further in-depth study. There is relatively little research on residual stress and fatigue life, and there is a lack of systematic microstructure–properties–process parameter relationships, which need future research. In addition, research on computer simulation is currently in a relatively immature state, and further studies on numerical simulation are needed to reveal the thermal coupling, material flow, and other behaviors introduced by the AFSD process, in order to better guide the optimization of process parameters in the design of print heads. At present, there is no very ideal computer aided design/manufacturing software available for AFSD, so there is an urgent need for software that can integrate 3D modeling and process parameter writing, which will greatly improve efficiency, reduce technical barriers, and enhance the competitiveness of AFSD. All of the above questions may be future research directions.

## Figures and Tables

**Table 1:** Mechanical properties of AA5083-H131 feedstock and AFSD deposit [34].

Material (Direction)	E (GPa)	YS (MPa)	UTS (MPa)	EL (%)
AA5083-H131 [34]	82.9 ± 0.9	273.7 ± 1.0	410 ± 6.1	0.15 ± 0.024
AA5083-H131(LD) [34]	70.8 ± 5.2	151.3 ± 1.7	431.3 ± 1.96	0.30 ± 0.005
AA5083-H131(BD) [34]	68.9 ± 5.9	157.7 ± 1.2	246.2 ± 45.9	0.08 ± 0.045

**Table 2:** Comparison of mechanical properties and grain size for WE43 magnesium alloys under different conditions [66,67].

Material (Direction)	YS (MPa)	UTS (MPa)	EL (%)	Average Grain Size (μm)
WE43-T5 [67]	265	335	19	13 ± 7
WE43 Underaged [67]	138	235	15	112 ± 55
WE43-T6 [67]	159	243	15	114 ± 58
WE43 AFSD (BD) [66]	200.8 ± 6.7	264.7 ± 10.8	11.0 ± 2.5	3.7 ± 3.3
WE43 AFSD (TD) [66]	230.0 ± 12.8	283.0 ± 6.1	11.7 ± 1.1	
WE43 AFSD (LD) [66]	213.8 ± 7.5	268.5 ± 3.4	13.8 ± 2.4	

**Table 3:** Quick reference of key conclusions.

Feedstock	Print Head	Key Conclusions
AA2014, Rod [9]	Consumable	Huge flash; rough top surface.
SS304, Rod [11]	Consumable	Poor interface bonding.
AA6061, Rod [14]	Non-consumable	Good interfacial bonding.
AA2024, Rod [15,16]	Non-consumable	Non-planar interface; fin and serration structures; material flow and shearing.
AA6061, Rod [17]	Non-consumable	No bonding when the layer thickness was too large.
WE43, Powder [18]	Non-consumable	Powder jammed in the print head; weaker mechanical properties than rod as feedstock.
AA5083, Chip [28]	Non-consumable	Increased UTS and fatigue life.
AA4043, Wire [29]	Non-consumable	Better macroscopic morphology.
AA6061, Wire [30]	Non-consumable	Improved the flexibility of AFSD.
IN625, Rod [31]	Non-consumable	Grain size refined.
AA2195, Rod [32]	Non-consumable	Superior mechanical properties at the top layer.
AA2219, Rod [33]	Non-consumable	Dissolution of θ′, replaced by θ.
AA5083, Rod [34,35]	Non-consumable	YS, UTS, and EL decreased in BD; improvement after compression deformation.
AA6061, Rod [36]	Non-consumable	Properties close to feedstock after heat treatment.
AA6061, Rod [38,39,40,41]	Non-consumable	The lower the rotation speed and the smaller the layer thickness, the finer the grain size.
AA6061, Rod [43,44]	Non-consumable	Location dependence of residual stress.
AA7075, Rod [51]	Non-consumable	Forging-like tensile properties after T74.
TiB_2_/AA7050, Rod [52]	Non-consumable	Evenly dispersed TiB_2_.
AA7075, Rod [53]	Non-consumable	As the amount of overlapping increased, the ductility of the material also enhanced.
AZ31, Rod [55,63]	Non-consumable	Enhanced the texture intensity, microstructures, and mechanical properties similar to forging ones.
GW83, Rod [64]	Non-consumable	Grain size and mechanical properties are not uniformly distributed across the width.
WE43, Rod [65,66]	Non-consumable	Decreased fatigue life under low-cycle conditions; performance under high-cycle conditions similar to forgings; better YS and UTS than underaged and T6 samples.
Ti64, Rod [68]	Non-consumable	EL of 7 ± 1%, YS of 1050 ± 25 MPa, and UTS of 1140 ± 20 MPa.
Ti64, Rod [69]	Non-consumable	“Basket weave” shape α-Ti in the prior β grains of 25–50 μm.
SS316, Rod [70]	Non-consumable	Formation of a gradient in twinning and martensitic phases following deformation.
DSS2507, Rod [71]	Non-consumable	High dependence on machinal properties.

## Data Availability

All data supporting the findings of this study are available within the paper and in references, further inquiries can be directed to the corresponding authors.
